# Stressful life events and their association with symptom severity and functioning in first-episode psychosis

**DOI:** 10.1038/s41537-026-00778-3

**Published:** 2026-06-26

**Authors:** Stefania Tognin, Mariia Moroz, Aljawharah Almuqrin, Maria Chiara Del Piccolo, Ryan Hammoud, Anna Georgiades, Andrea Mechelli

**Affiliations:** 1 Department of Psychosis Studies, Institute of Psychiatry, Psychology, and Neuroscience (IoPPN), King’s College London, London, UK; 2 Department of Health Sciences, College of Health and Rehabilitation Sciences, Princess Nourah bint Abdulrahman University, Riyadh, Saudi Arabia; 3 Brent Early Intervention Service, 27-29 Fairlight Avenue, CNWL, NHS Foundation Trust, London, NW10 8AL UK

**Keywords:** Schizophrenia, Human behaviour

## Abstract

Stressful life events (SLEs) are a risk factor for psychosis; however, evidence on the impact of SLEs in individuals with first-episode psychosis (FEP) is limited. The current study draws on longitudinal data from the social mind study (ISRCTN85485447) and examines whether SLEs predict subsequent symptom severity, functioning, and relapse among individuals with first-episode psychosis (FEP). We hypothesised that greater exposure to SLEs at baseline would be associated with poorer clinical outcomes at follow-up. Data from 265 FEP participants across 10 sites in the United Kingdom were examined to investigate the associations between baseline SLEs and changes in symptom severity, functioning, and relapse over a 1-year follow-up. Statistical models were adjusted for age, sex, ethnicity, and site. Greater baseline SLE exposure predicted more severe general psychopathology both across assessment timepoints (*p* < 0.05) and at 12 months (*p* < 0.05). Baseline SLEs predicted poorer functioning (measured using GAF symptoms and disability) across assessment timepoints (*p* < 0.05). Baseline SLE exposure was not associated with increased odds of relapse. In conclusion, this study shows that FEP individuals who were exposed to SLEs prior to baseline exhibited more severe general psychopathology and poorer functioning. SLEs assessment should be integrated into early psychosis care and inform early intervention strategies addressing social stress.

## Background

Stressful life events (SLEs), defined as discrete experiences that require substantial psychological adjustment and are perceived as threatening or disruptive to an individual’s usual functioning (e.g. bereavement, interpersonal loss, or financial hardship), have long been recognised as a major environmental risk factor for mental health outcomes, particularly the onset and progression of psychosis^1^. The diathesis–stress model provides a theoretical framework, suggesting that psychotic experiences and disorders emerge when individuals with underlying vulnerabilities are exposed to acute stress^2^. Extending this perspective, network analysis has shown that life-event burden, the cumulative impact of stressful events, is strongly associated with psychotic symptoms primarily through its impact on general psychopathology, in particular, broad emotional distress such as depression, anxiety, guilt, and tension^3^. Together, these findings indicate that psychosocial stress occurring shortly before or early in the course of illness may shape both symptom presentation and functioning.

Empirical evidence further supports this link. A recent systematic review reported that, among individuals with a diagnosed psychotic disorder, psychosocial stress triggered by exposure to SLEs was associated with an increased risk of relapse^4^. Similarly, in populations at clinical high risk for psychosis (CHR-P), exposure to SLEs has been associated with an elevated risk of transition to psychosis^5^, greater symptom severity, and poorer functioning^6^. Despite these findings across both high-risk and established-diagnosis groups, the extent to which SLEs influence clinical outcomes in the early stages of psychosis remains unclear.

Individuals experiencing a first episode of psychosis (FEP), defined as the first occurrence of psychotic symptoms^7^, are an important target population. The first couple of years following psychosis onset constitute a critical period that determines long-term outcomes^8–10^. For instance, a longer duration of untreated psychosis has been linked to worsening of symptoms, functioning, and a more severe illness course^11^. Notably, individuals with FEP are approximately three times more likely to have been exposed to SLEs in the year preceding psychosis onset compared to the general population^12^. Beyond being at an elevated risk for such exposure, a 2-year prospective study using advanced causal inference methods revealed that, in patients with FEP, exposure to SLEs increased the risk of relapse, independent of shared genetic and environmental factors^13^. Altogether, these findings highlight the importance of exploring the impact of SLEs on clinical outcomes in FEP, as this may inform targeted early intervention strategies.

However, research findings are mixed. Some studies report inconclusive findings and point to variability in the impact of SLEs on FEP clinical outcomes^14^, while others report no impact of life events on psychotic symptoms altogether^15^. Inconsistencies likely reflect important methodological and conceptual differences across studies. For instance, ref. ^13^ used a prospective design in a first-episode psychosis (FEP) cohort with clear temporal ordering of events, demonstrating that stressful life events temporally precede and increase the risk of relapse, supporting a predominantly unidirectional and near-causal interpretation. In contrast, ref. ^14^, drawing on repeated assessments over a 10-year period, reported bidirectional associations between life events and symptom course, whereby stressful experiences predict symptom exacerbation, but symptom severity also increases subsequent exposure to stress, highlighting dynamic feedback processes. Parmigiani et al. ^15^, using a cross-sectional design across mixed diagnostic groups, found no clear association between life events and psychotic symptoms, instead emphasising perceived stress and basic symptoms as markers of underlying vulnerability, without establishing temporal direction. These studies further differ in outcome definitions (relapse vs continuous symptom trajectories vs basic symptoms), stress measurement (objective life events vs perceived stress), and sample composition (FEP-specific vs transdiagnostic), limiting direct comparability. Taken together, these differences suggest that mixed findings in the literature may arise because studies capture different levels of the stress–psychosis relationship, ranging from acute triggering of relapse to long-term reciprocal dynamics, to subjective stress appraisal. To our knowledge, no studies to date have explored how SLEs, measured specifically at baseline, thereby capturing exposure prior to (i.e. the year before) or during the early illness stage, predict longitudinal clinical outcomes in individuals with FEP. Addressing this would not only advance the understanding of prognostic factors in FEP but would also enable the identification of potential treatment targets.

The current study aimed to examine whether recent SLE exposure at baseline would predict symptom severity, functioning and psychosis relapse over 12 months within a FEP sample using a longitudinal dataset from the Social Mind study (https://www.isrctn.com/ISRCTN85485447).

We hypothesised that higher levels of baseline stressful life events (SLEs) would be associated with poorer clinical outcomes at follow-up. Specifically, we expected that individuals reporting more baseline SLEs would show greater symptom severity at 12 months (Hypothesis 1) and across all assessment points (Hypothesis 2). We further hypothesised that higher baseline SLEs would predict lower functioning at 12 months (Hypothesis 3) and across all assessment points (Hypothesis 4). Finally, we anticipated that higher baseline SLEs would be associated with increased odds of relapse at 12 months (Hypothesis 5). All analyses controlled for age, sex, ethnicity, and site to account for potential confounding influences.

## Methods

### Participants

The Social Mind study is an observational, longitudinal and multi-centre study. Participants ranged from 18 to 40 years of age were recruited across 10 sites. The sites included Cardiff and Vale University—University Hospital of Wales, South West London and St George’s Mental Health NHS Trust, and the following NHS Foundation Trusts: South London and Maudsley, East London, North East London, Oxford Health, Sheffield Health and Social Care, Surrey and Borders Partnership, Black Country Healthcare, Central and North West London. Potential participants were approached by their care team and asked whether they were interested in taking part in the Social Mind study (https://www.isrctn.com/ISRCTN85485447). Those who expressed interest were provided with an information sheet, and informed consent was subsequently obtained by a member of the research team. As only individuals who had consented to be contacted were approached, no data were collected on the number or characteristics of those who declined participation.

Participants were eligible for the study if they could fully comprehend the activities/requirements related to the study and had experienced a FEP in the last 24 months. Diagnosis of a psychotic disorder was confirmed using the Structured Clinical Interview for DSM Disorders. The exclusion criteria for all participants included (1) refusal to complete the clinical interview; (2) inability to comprehend the study or provide consent; (3) estimated IQ <70; (4) >24 months between psychosis onset and study entry; (5) currently sectioned or severely unwell; (6) experienced more than one previous episode of psychosis. Participants were followed up for a 1-year period and were assessed at four timepoints: baseline, 4 months, 8 months and 12 months. Assessments were performed by trained raters.

When participants did not return for follow-up appointments, or when not all data could be gathered during the interview, clinical records were used to collect information about relapse. The authors assert that all procedures contributing to this work comply with the ethical standards of the relevant national and institutional committees on human experimentation and with the Helsinki Declaration of 1975, as revised in 2013. All procedures involving human subjects/patients were approved by the Research Ethics Committee (20/LO/0331 /IRAS project ID: 278399). All participants provided written informed consent. As this study represents a secondary analysis of an existing longitudinal first-episode psychosis cohort, the sample size was determined by the parent study rather than an a priori power calculation for the present hypotheses.

### Materials

The materials described below are relevant to the current study and were administered at baseline and subsequent follow-up assessments. Additional information is included in the Supplementary Materials.

#### Sociodemographic data

A case report file was used to collect baseline demographic data, including age, sex, ethnicity and recruiting site.

#### Stressful life events

The list of threatening life experiences (LTE) questionnaire^16^ was used to assess exposure to SLEs at baseline. The LTE is a widely used 12-item self-report inventory designed to capture major life events associated with substantial long-term contextual threat, such as bereavement, serious illness or injury, unemployment, or major financial difficulties. It was developed as a brief and practical alternative to more resource-intensive interview-based measures, such as the life events and difficulties schedule (LEDS), while retaining a focus on events most strongly linked to psychiatric risk.

The questionnaire is administered as a checklist referring to a defined time period (i.e. the past 12 months), and responses are summed to provide an overall index of recent adversity. The measure has demonstrated good psychometric properties, including high test–retest reliability and strong validity when compared with interview-based assessments and informant reports^17^.

#### Outcome measures

The Positive and Negative Syndrome Scale^18^ was used to assess symptom severity. Participants were interviewed on 30 items divided into three symptom subscales: positive symptoms (seven items), negative symptoms (seven items), and general psychopathology (16 items). Each item was rated on a seven-point Likert scale ranging from 1 = ‘absent’ to 7 = ‘extreme’, with higher scores indicating greater symptom severity. A total PANSS score for each participant was calculated as the sum of all symptom subscales.

The global assessment of functioning (GAF) scale^19^ was used to assess functioning, divided into symptom and disability subscales, as seen in previous studies^6,20^. The GAF disability scale resembles the well-established Social and Occupational Functioning Assessment Scale^21^. Both subscales ranged from 0 to 100, with higher scores indicating better functioning within the previous month.

Relapse status indicated a return or an exacerbation of psychotic symptoms following a period of remission. This information was collected at each assessment based on the online clinical interview and confirmed through clinical records. For the purpose of statistical analyses, relapse was operationalised as a single binary outcome indicating whether a participant had relapsed at any point during the 12-month assessment period following baseline.

### Statistical analysis

All analyses were performed in Stata version 18. The statistical significance level was set at *p* < 0.05 (two-tailed). While the study aimed at assessing participants at regular time intervals, there were occasions when subsequent follow-up assessments did not match the intended follow-up date, or the data were not collected. The statistical linear mixed models (LMMs) used in the study accounted for deviations in follow-up timing and missing data, allowing inclusion of all available observations per participant.

For cross-sectional analyses, Stata default listwise deletion was applied. Thus, only participants with complete data on relevant variables were included in each model, and sample sizes varied across analyses depending on missing data for each variable (Supplementary Table S1).

For longitudinal models, maximum likelihood estimation was used, which allowed for the inclusion of all available observations per participant, maximising data availability (Supplementary Tables S2 to S4).

Baseline demographic characteristics of participants, such as age, sex, ethnicity, site, and medication usage, were analysed (Table 1) and were included as potential confounders for subsequent analyses.

**Table 1:** Baseline demographic characteristics of participants.

Variable	
Age (years), mean (SD)	26.07 (5.28)
Sex at birth, *n* (%)	
Male	149 (56.23)
Female	116 (43.77)
Ethnicity, *n* (%)	
White	98 (36.98)
Black	60 (22.64)
East Asian	6 (2.26)
Asian Indian	20 (7.55)
Middle Eastern	7 (2.64)
Other	74 (27.92)
Medication, *n* (%)	
Antipsychotic	219 (79.93)
Other	17 (6.20)
None	30 (10.95)

#### Cross-sectional analysis

To provide an initial overview of the relationship between the baseline LTE and each outcome variable, a series of simple linear regressions was run. For each model, the baseline LTE was entered as the exposure variable, and each of the 12-month follow-up continuous outcome measures (PANSS total, PANSS positive, PANSS negative, PANSS general, GAF disability and GAF symptoms) served as the dependent variable. These models were first run unadjusted and then adjusted for age, sex, ethnicity, site and medication usage.

#### Longitudinal analysis

LMMs were used to examine the longitudinal association between baseline LTE and continuous outcome measures (PANSS total, PANSS positive, PANSS negative, PANSS general, GAF disability and GAF symptoms) across assessment timepoints. Assessment timepoint was included as a categorical fixed effect, allowing outcome scores at each assessment to be compared without assuming a linear change over time. LMMs include both fixed effects and random effects, which account for correlations between repeated measurements within each participant.

For each outcome, the best-fitting random-effects structure was selected by comparing a random intercepts-only model to a model including both random intercepts and slopes, using Likelihood Ratio Tests (LRTs). This approach ensured that the final model structure for each outcome was statistically appropriate. Additionally, LMMs accommodate missing data under the missing-at-random (MAR) assumption^22^. Sample sizes varied across assessment timepoints due to incomplete follow-up data and attrition, with the number of participants at each timepoint is reported in the Supplementary Materials (Supplementary Tables S1–S5). Under the MAR assumption, missingness is assumed to be related to observed data rather than unobserved outcomes, conditional on the variables included in the model.

First, models were fitted with assessment timepoint (months from baseline) as the sole predictor to examine the overall trajectory of the sample. Baseline LTE was then added to evaluate its main effect across timepoints. Adjusted models controlled for age, sex, ethnicity, site and medication usage to account for potential confounding.

#### Logistic regression

To assess whether baseline LTE predicted relapse within 12 months, logistic regression was conducted with relapse status as a binary outcome. An unadjusted model was run first, followed by a model adjusting for age, sex, ethnicity, site and medication usage as potential confounds.

## Results

About 274 participants were recruited between December 2020 and December 2023. In the current study, we included a subset of 265 participants for whom baseline SLE data were available. Demographic characteristics of participants collected at baseline are summarised in Table 1.

Analytic sample sizes for each model are provided in Supplementary Tables.

### Cross-sectional regressions

Baseline LTE significantly and positively predicted PANSS total at 12 months (*b* = 1.03, *p* = 0.032). This association remained significant after adjusting for age, sex at birth, ethnicity, site, and medication usage (*b* = 1.06, *p* = 0.032). At 12 months, baseline LTE was not significantly associated with either GAF symptoms or GAF disability, in both unadjusted and adjusted models (Table 2 and Supplementary Table S6). When analysing PANSS domains separately, baseline LTE were not significantly associated with PANSS positive or negative symptoms at 12 months in either unadjusted or adjusted models. However, there was a significant positive significant association between baseline LTE and PANSS general symptoms at 12 months, both in the unadjusted model (*b* = 0.77, *p* = 0.004) and the adjusted model (*b* = 0.77, *p* = 0.005).

**Table 2:** Adjusted cross-sectional associations between baseline LTE total scores and continuous outcomes at 12 months.

			95% CI		
Outcome	*b*	SE	LL	UL	*p*
PANSS	1.05	0.49	0.09	2.02	0.032*
PANSS-P	0.09	0.13	−0.17	0.35	0.482
PANSS-N	0.14	0.18	−0.20	0.49	0.413
PANSS-G	0.77	0.27	0.23	1.32	0.005*
GAF-S	−1.02	0.80	−2.61	0.56	0.203
GAF-D	−1.45	0.79	−3.01	0.10	0.067

These models were adjusted for age at baseline, sex, ethnicity, and site; the analysis only included participants for whom LTE baseline data were available. Unadjusted results are in Supplementary Table S6.

*LTE* list of threatening experiences, *PANSS* positive and negative syndrome scale, *PANSS-P* positive and negative syndrome scale—positive symptoms, *PANSS-N* positive and negative syndrome scale —negative symptoms, *PANSS-G* positive and negative syndrome scale—general symptoms, *GAF-S* global assessment of functioning—symptoms, *GAF-D* global assessment of functioning—disability.

*p < 0.05.

### Longitudinal mixed models

Model comparison using LRTs indicated that adding random slopes for assessment timepoint improved model fit for both GAF symptoms (*X*^*2*^(1) = 18.16, p < 0.001) and GAF disability (*X*^*2*^(1) = 11.29, p < 0.001), but not for PANSS total (*LR X*^*2*^(1) = 0.23, *p* = 0.633). Longitudinal PANSS and GAF trajectories over 4-, 8- and 12 months are available in Supplementary Tables S7 and S8.

Higher baseline LTE was significantly associated with greater PANSS total when controlling for time (*b* = 0.76, *p* = 0.014), and this remained significant after adjusting for age, sex at birth, ethnicity, site, and medication usage (*b* = 0.74, *p* = 0.021). When analysing PANSS domains separately, it emerged that higher baseline LTE was significantly associated with greater PANSS general symptoms score when controlling for time (*b* = 0.63, *p* < 0.001), and this remained significant after adjusting for age, sex at birth, ethnicity, site, and medication usage (*b* = 0.63, <0.001). For PANSS positive symptoms, this association was not statistically significant in the unadjusted model *(p* = 0.138) and the adjusted model (*p* = 0.180), as well as for PANSS negative symptoms in the unadjusted model *(p* = 0.925) and the adjusted model (*p* = 0.911). For both GAF disability and GAF symptoms, the associations were not statistically significant in unadjusted models (p = 0.057 and p > 0.05, respectively), but became significant after adjusting for age, sex at birth, ethnicity, site, and medication usage (disability: *b* = −1.10, *p* = 0.022; symptoms: *b* = −1.14, *p* = 0.015). Results are presented in Table 3.

**Table 3:** Adjusted longitudinal mixed model associations between baseline LTE total scores and continuous outcomes.

			95% CI		
Outcome	*b*	SE	LL	UL	*p*
PANSS	0.74	0.32	0.11	1.38	0.021*
PANSS-P	0.13	0.09	−0.06	0.31	0.180
PANSS-N	−0.01	0.11	−0.22	0.19	0.911
PANSS-G	0.63	0.18	0.28	0.99	<0.001**
GAF-S	−1.14	0.47	−2.05	−0.22	0.015*
GAF-D	−1.10	0.48	−2.04	−0.16	0.022*

Time was modelled as a categorical predictor with baseline as the reference. All models were adjusted for age at baseline, sex, ethnicity, and site. Unadjusted results are in Supplementary Table S8.

*PANSS* positive and negative syndrome scale, *PANSS-P* positive and negative syndrome scale—positive symptoms, *PANSS-N* positive and negative syndrome scale—negative symptoms, *PANSS-G* positive and negative syndrome scale—general symptoms, *GAF-S* global assessment of functioning—symptoms, *GAF-D* global assessment of functioning—disability.

*p < 0.05

**p < 0.001

### Logistic regression

Relapse occurred in 50/190 participants within 12 months. Baseline LTE did not significantly predict relapse status in either the unadjusted model or the model adjusting for age, sex at birth, ethnicity, site, and medication usage. In the adjusted model, baseline LTE was also not significantly associated with relapse (*OR* = 1.09, 95% *CI* [0.91, 1.30], *p* = 0.355). Results are available in Supplementary Table S9.

## Discussion

The current study examined whether baseline SLE exposure predicted symptom severity, functioning, and relapse over 12 months in individuals with FEP.

Results showed that higher baseline SLEs predicted more severe symptoms both at 12-month follow-up and across all assessment points (Hypotheses 1 and 2). However, contrary to our hypothesis, baseline SLEs did not predict functioning at 12 months (Hypothesis 3). The predicted effect of SLEs on functioning was supported, with GAF symptoms and GAF disability, being positively associated with functioning across timepoints (Hypothesis 4). Finally, contrary to our hypothesis, higher baseline SLEs did not predict an increase in the odds of relapse within 12 months (Hypothesis 5).

Our finding that higher baseline SLEs were predictive of worse symptom severity at 12-month follow-up is, in part, consistent with previous studies^14^, which found that negative life events predicted positive symptoms, and a network analysis^3^, which showed that higher recent life-event burden was associated with worse general psychopathology symptoms. This was further supported by our finding that participants with higher baseline SLEs exhibited higher symptom severity over the 12-month period, indicating a sustained effect, consistent with similar findings in CHR-P samples^6^. Notably, in our sample, SLEs were more strongly associated with PANSS general symptoms than with positive or negative psychotic symptoms. One possible explanation is that SLEs exert a broader, non-specific impact on domains captured by the PANSS general subscale, such as anxiety, depression, guilt, tension, and poor impulse control, which are more directly and consistently sensitive to environmental stressors^3^. These general symptoms may reflect heightened emotional distress and dysregulation arising from stress exposure, as well as alterations in stress-response systems (e.g. HPA-axis activation), which are less specific to psychosis but central to overall clinical severity^23^.

Contrary to our prediction, baseline exposure to SLEs did not predict functioning at 12 months. However, greater baseline SLEs were associated with worse functioning on both GAF symptoms and GAF disability subscales across follow-up assessments in the longitudinal models. This pattern suggests that SLEs may be more closely related to fluctuations in functioning over time rather than outcomes at a single distal timepoint. The finding is theoretically coherent: the GAF symptoms subscale captures recent symptom burden (e.g. psychotic features, anxiety and suicidality), which may be more sensitive to stress-related processes, whereas GAF disability reflects social and occupational functioning, which tends to evolve more gradually and is likely influenced by broader, more stable factors. The absence of an association at 12 months is likely attributable to several methodological considerations. First, SLEs were measured only at baseline (covering the prior 12 months) and not reassessed, meaning that their temporal proximity to functioning outcomes decreases over time, potentially attenuating effects at later timepoints. Second, there is a mismatch in measurement windows, with SLEs indexing past-year exposure and GAF reflecting functioning over the preceding month, a discrepancy that is greatest at the 12-month assessment. Third, the longitudinal mixed models draw on repeated observations and therefore have greater statistical power than single timepoint analyses, which may be more susceptible to missing data. Finally, the GAF is a relatively coarse, single-item measure; more comprehensive tools such as the WHODAS 2.0 may be better suited to capturing domain-specific functional changes and clarifying how SLEs impact different aspects of functioning over time^24^. The difference in the best-fitting random-effects structures for PANSS and GAF outcomes may point to differences in how stress exposure relates to symptoms compared to functioning over time. For PANSS total, adding random slopes for assessment timepoint did not improve model fit, suggesting limited between-participant variability in the rate of symptom change over time. This indicated a more consistent sample-level relationship between stressful life events and symptom severity. In contrast, random-slope models provided a better fit for both GAF domains, indicating greater heterogeneity in individual functional trajectories. This could reflect the more context-dependent nature of functioning, which is likely more sensitive to social, occupational and environmental factors in addition to symptom severity and stress exposure.

Contrary to our predictions, higher baseline SLEs did not significantly increase the odds of relapse within 12 months. This contrasts with previous evidence in psychotic disorder populations^4,13^ and CHR-P samples^5^, which has generally reported positive associations between life events and relapse. However, our findings are not necessarily inconsistent with this literature. For example, Bhattacharyya et al. ^13^ reported a 2.6-fold increase in relapse hazard over 2 years and a 36% increase in hazard per additional post-onset SLEs, indicating a dose–response relationship. Several methodological differences may help explain the comparatively attenuated effect in our study. First, SLEs were assessed for the 12 months prior to baseline, which could have occurred up to two years after FEP onset, potentially diluting temporal proximity between stress exposure and relapse. In contrast, prior studies focused specifically on SLEs occurring after onset and closer to relapse events. Second, our use of a binary relapse outcome within a fixed 12-month follow-up period may have reduced sensitivity to variation in relapse timing and frequency, whereas survival models (as used by Bhattacharyya et al.) capture both the occurrence and timing of relapse and are better suited to detecting dynamic risk processes. Additionally, it is possible that SLEs exert a more indirect or cumulative influence on relapse risk, for example through their sustained association with general psychopathology, rather than as acute proximal triggers captured within a fixed observation window. Taken together, these differences likely contribute to the smaller effect observed here, while the broader pattern of findings remains compatible with a role for SLEs in shaping relapse vulnerability following FEP.

### Limitations and strengths

We note several limitations warranting consideration. Firstly, SLEs were measured once at baseline (12-month recall) with an unweighted checklist that recorded occurrence but not severity, duration, or perceived impact. Because stressful events were assessed at baseline only, our measure reflects pre-study exposure. Any additional events during follow-up, if unrelated to outcomes, would likely lead to more conservative (i.e. attenuated) estimates of the associations. The recall window may also have mixed pre-onset and early-onset exposures, complicating temporal interpretation. Future work should code events as independent (unlikely to be influenced by the individual’s behaviour e.g. bereavement due to illness) or interpersonal (shaped by social interactions or illness-related behaviours e.g. relationship breakdown), as seen in previous studies^6,25,26^, and re-assess SLEs at each wave, allowing for more robustness and stronger temporal inference. This distinction is particularly important for causal inference as interpersonal events may partly reflect the effects of emerging symptoms, complicating the interpretation of their role as causal risk factors. Secondly, relapse was analysed as a fixed 12-month binary outcome, omitting information on timing and multiple relapses. A survival analysis, as employed in research by ref. ^13^, would address this. Our LMM analyses relied on the MAR assumption and did not apply multiple imputation for covariates or inverse-probability weighting for missing data. If there was selective attrition, where participants with higher SLE exposure, worse symptoms, or functioning at baseline were less likely to complete later assessments, estimates may have been attenuated and interaction effects under-detected. Thirdly, participants were recruited via NHS Early Intervention Services, reflecting treated rather than population incidence. Only individuals who had consented to be contacted were approached by the research team, and data were collected only from those who consented, precluding assessment of non-participants. Individuals in acute crisis were also less likely to be approached or to participate, potentially introducing further selection bias. Finally, no formal a priori power calculation was conducted for the present analyses, as the sample size was determined by the parent study. Although the sample is comparable to, or larger than, many longitudinal studies in first-episode psychosis, it is possible that some analyses, particularly those examining relapse outcomes, were underpowered to detect small effects.

This study also has several strengths. Firstly, a multi-site and diverse sample improved heterogeneity and strengthened external validity. Secondly, the prospective, four-wave follow-up across 12 months captured individual trajectories rather than static cross-sections, enabling robust estimation of change over time and associations with SLEs. Thirdly, while points of refinement in measurement choices remain, the use of validated instruments ensured construct validity, facilitated comparability with prior work, and supports future replication. Finally, the analyses used LMM models with pre-specified confounder adjustments. These models account for within-participant dependence, appropriately accommodate irregular follow-ups and incomplete observations under the MAR assumption, and maximise statistical power and precision without sacrificing interpretability.

## Conclusion

Overall, our findings suggest that exposure to SLEs has important clinical relevance for individuals with first-episode psychosis, particularly in relation to symptom severity rather than relapse risk. Higher levels of SLEs were associated with greater overall symptom burden over time, with a more pronounced relationship with general psychopathology symptoms, indicating a sustained impact of stress on broader affective and distress-related domains. These findings highlight the potential value of assessing recent SLEs at intake and during follow-up to better characterise clinical course and identify individuals at risk of poorer symptomatic outcomes. However, the lack of association with relapse suggests that SLEs may not operate as proximal triggers of relapse within a limited follow-up window, but rather as factors shaping ongoing symptom severity. Future research should examine whether interventions such as CBT for psychosis, particularly those targeting stress appraisal and coping, may be especially beneficial for individuals with higher SLE exposure.

## Supplementary information

Supplementary Tables S1 to S9

## Data Availability

The data generated and analysed for the current study are not publicly available in accordance with ethical and institutional guidelines, but de-identified data may be made available from the corresponding author on reasonable request.
