# Supplementary material for: Stressful life events and their association with symptom severity and functioning in first-episode psychosis
Source: Schizophrenia (Heidelb). 2026 Jun 26;12(1):79. doi: 10.1038/s41537-026-00778-3 (PMC13672798; doi:10.1038/s41537-026-00778-3)
Supplement: Supplementary file 1 [file 41537_2026_778_MOESM1_ESM.docx]

**Supplementary Materials**

| **Table S1.**  *Sample size in adjusted and unadjusted cross-sectional analyses* | |
| --- | --- |
| Outcome at 12 months follow-up | n |
| PANSS | 164 |
| PANSS-P | 165 |
| PANSS-N | 165 |
| PANSS-G | 164 |
| GAF symptoms | 173 |
| GAF disability | 173 |
| Participants included have LTE data recorded at baseline. | |

| **Table S2.**  *Sample size in adjusted and unadjusted longitudinal analyses* | | | | | | |
| --- | --- | --- | --- | --- | --- | --- |
| Assessment | PANSS, n | PANSS-P, n | PANSS-N, n | PANSS-G, n | GAF symptoms, n | GAF disability, n |
| Baseline | 264 | 264 | 264 | 264 | 262 | 261 |
| 4 months | 193 | 194 | 194 | 194 | 198 | 197 |
| 8 months | 172 | 172 | 172 | 172 | 178 | 178 |
| 12 months | 164 | 165 | 165 | 164 | 173 | 173 |
| Participants included have LTE data recorded at baseline. | | | | | | |

| **Table S3.**  *Sample size in adjusted and unadjusted longitudinal analyses* | | | | | | |
| --- | --- | --- | --- | --- | --- | --- |
| Assessment | PANSS, n | PANSS-P, n | PANSS-N, n | PANSS-G, n | GAF symptoms, n | GAF disability, n |
| Baseline | 262 | 262 | 262 | 262 | 260 | 259 |
| 4 months | 192 | 193 | 193 | 193 | 197 | 196 |
| 8 months | 171 | 171 | 171 | 171 | 177 | 177 |
| 12 months | 164 | 165 | 165 | 164 | 173 | 173 |
| Participants included have LTE data recorded at baseline. | | | | | | |

| **Table S4.**  *Sample size in adjusted and unadjusted longitudinal analyses* | | | | | | |
| --- | --- | --- | --- | --- | --- | --- |
| Assessment | PANSS, n | PANSS-P, n | PANSS-N, n | PANSS-G, n | GAF symptoms, n | GAF disability, n |
| Baseline | 264 | 264 | 264 | 264 | 261 | 260 |
| 4 months | 194 | 195 | 195 | 195 | 199 | 198 |
| 8 months | 173 | 173 | 173 | 173 | 179 | 179 |
| 12 months | 166 | 167 | 167 | 166 | 175 | 175 |
| Participants included have LTE data recorded at baseline. | | | | | | |

| **Table S5.**  *Sample size in unadjusted LMMs: associations between assessment timepoint (predictor) and continuous outcome variables* | | | | | | |
| --- | --- | --- | --- | --- | --- | --- |
| Assessment | PANSS, n | PANSS-P, n | PANSS-N, n | PANSS-G, n | GAF symptoms, n | GAF disability, n |
| Baseline | 266 | 266 | 266 | 266 | 263 | 262 |
| 4 months | 195 | 196 | 196 | 196 | 200 | 199 |
| 8 months | 174 | 174 | 174 | 174 | 180 | 180 |
| 12 months | 166 | 167 | 167 | 166 | 175 | 175 |
| Participants included have LTE data recorded at baseline. | | | | | | |

| **Table S6.**  *Unadjusted cross-sectional associations between baseline LTE total scores and continuous outcomes at 12 months* | | | | | |
| --- | --- | --- | --- | --- | --- |
|  |  |  | 95% CI | |  |
| Outcome | *b* | SE | LL | UL | *p* |
| PANSS | 1.03 | 0.48 | 0.09 | 1.97 | .032 |
| PANSS-P | 0.10 | 0.13 | -0.15 | 0.36 | .431 |
| PANSS-N | 0.12 | 0.17 | -0.22 | 0.45 | .497 |
| PANSS-G | 0.77 | 0.27 | 0.24 | 1.29 | **.004*** |
| GAF-S | -0.10 | 0.78 | -2.54 | 0.55 | .204 |
| GAF-D | -1.37 | 2.13 | -2.89 | 0.16 | .079 |
| **p* < .05  *Note.* LTE, List of Threatening Experiences; PANSS, Positive and Negative Syndrome Scale; PANSS-P, Positive and Negative Syndrome Scale – positive symptoms; PANSS-N, Positive and Negative Syndrome Scale – negative symptoms; PANSS-G, Positive and Negative Syndrome Scale – general symptoms; GAF-S, Global Assessment of Functioning - symptoms; GAF-D, Global Assessment of Functioning – disability. The analysis only included participants for whom LTE baseline data was available. | | | | | |

| **Table S7.**  Adjusted longitudinal mixed models of continuous outcomes across assessment timepoints | | | | | | |
| --- | --- | --- | --- | --- | --- | --- |
| Outcome | Predictor | *b* | SE | 95% CI | | *p* |
|  |  |  |  | LL | UL |  |
| PANSS | 4 months | -1.50 | 0.82 | -3.10 | 0.10 | .067 |
|  | 8 months | -1.91 | 0.85 | -3.58 | -0.23 | **.026*** |
|  | 12 months | -2.95 | 0.87 | -4.66 | -1.24 | **.001**** |
| PANSS-P | 4 months | -0.04 | 0.23 | -0.49 | 0.42 | .873 |
|  | 8 months | -0.25 | 0.24 | -0.72 | 0.23 | .311 |
|  | 12 months | -0.46 | 0.25 | -0.94 | 0.02 | .063 |
| PANSS-N | 4 months | -0.38 | 0.29 | -0.95 | 0.18 | .187 |
|  | 8 months | -0.58 | 0.30 | -1.18 | 0.01 | .053 |
|  | 12 months | -0.82 | 0.31 | -1.42 | -0.22 | **.008*** |
| PANSS-G | 4 months | -1.20 | 0.48 | -2.13 | -0.27 | **.011*** |
|  | 8 months | -1.12 | 0.50 | -2.10 | -0.15 | **.024*** |
|  | 12 months | -1.81 | 0.51 | -2.81 | -0.82 | **<.001**** |
| GAF-S | 4 months | 1.76 | 1.02 | -0.24 | 3.77 | .085 |
|  | 8 months | 1.10 | 1.12 | -1.10 | 3.29 | .328 |
|  | 12 months | 1.92 | 1.22 | -0.47 | 4.31 | .116 |
| GAF-D | 4 months | 1.26 | 1.02 | -0.74 | 3.27 | .217 |
|  | 8 months | 1.82 | 1.11 | -0.35 | 3.99 | .099 |
|  | 12 months | 2.16 | 1.19 | -0.17 | 4.49 | .069 |
| **p* < .05  ***p* < .001  Note. b-LTE, baseline List of Threatening Experiences; PANSS, Positive and Negative Syndrome Scale; PANSS-P, Positive and Negative Syndrome Scale – positive symptoms; PANSS-N, Positive and Negative Syndrome Scale – negative symptoms; PANSS-G, Positive and Negative Syndrome Scale – general symptoms; GAF-S, Global Assessment of Functioning - symptoms; GAF-D, Global Assessment of Functioning – disability. Time was modelled as a categorical predictor with baseline as the reference. All models were adjusted for age at baseline, sex, ethnicity, and site. | | | | | | |

| **Table S8.**  *Unadjusted longitudinal mixed models* | | | | | | |
| --- | --- | --- | --- | --- | --- | --- |
| Outcome | Predictor | *b* | SE | 95 %CI | | *p* |
|  |  |  |  | LL | UL |  |
| PANSS | 4 months | -1.54 | 0.81 | -3.13 | 0.06 | .059 |
|  | 8 months | -2.08 | 0.85 | -3.74 | -0.42 | **.014*** |
|  | 12 months | -3.12 | 0.86 | -4.81 | -1.43 | **<.001**** |
|  | b-LTE | 0.76 | 0.31 | 0.16 | 1.37 | **.014*** |
| PANSS-P | 4 months | -0.07 | 0.23 | -0.52 | 0.38 | .769 |
|  | 8 months | -0.31 | 0.24 | -0.78 | 0.16 | .195 |
|  | 12 months | -0.52 | 0.24 | -0.99 | -0.04 | **.033*** |
|  | b-LTE | 0.13 | 0.09 | -0.04 | 0.31 | .138 |
| PANSS-N | 4 months | -0.39 | 0.29 | -0.95 | 0.17 | .174 |
|  | 8 months | -0.60 | 0.30 | -1.18 | -0.01 | **.047*** |
|  | 12 months | -0.84 | 0.30 | -1.43 | -0.24 | **.006*** |
|  | b-LTE | 0.01 | 0.10 | -0.19 | 0.21 | .925 |
| PANSS-G | 4 months | -1.21 | 0.47 | -2.13 | -0.28 | **.011*** |
|  | 8 months | -1.23 | 0.49 | -2.20 | -0.26 | **.013*** |
|  | 12 months | -1.91 | 0.50 | -2.89 | -0.93 | **<.001**** |
|  | b-LTE | 0.63 | 0.17 | 0.29 | 0.96 | **<.001**** |
| GAF-S | 4 months | 1.80 | 1.02 | -0.21 | 3.80 | .079 |
|  | 8 months | 1.17 | 1.11 | -1.01 | 3.35 | .293 |
|  | 12 months | 2.05 | 1.21 | -0.32 | 4.42 | .090 |
|  | b-LTE | -0.78 | 0.46 | -1.68 | 0.12 | .091 |
| GAF-D | 4 months | 1.25 | 1.03 | -0.75 | 3.25 | .222 |
|  | 8 months | 1.77 | 1.10 | -0.38 | 3.93 | .107 |
|  | 12 months | 2.03 | 1.17 | -0.28 | 4.34 | .085 |
|  | b-LTE | -0.89 | 0.47 | -1.80 | 0.02 | .057 |
| **p* < .05  ***p* < .001  Note. b-LTE, baseline List of Threatening Experiences; PANSS, Positive and Negative Syndrome Scale; PANSS-P, Positive and Negative Syndrome Scale – positive symptoms; PANSS-N, Positive and Negative Syndrome Scale – negative symptoms; PANSS-G, Positive and Negative Syndrome Scale – general symptoms; GAF-S, Global Assessment of Functioning - symptoms; GAF-D, Global Assessment of Functioning – disability. Time was modelled as a categorical predictor with baseline as the reference. | | | | | | |

| **Table S9.**  *Logistic regression models examining the association between baseline LTE total score and relapse within 12 months* | | | | | | |
| --- | --- | --- | --- | --- | --- | --- |
| Model | n | Relapses, n | OR | 95% CI | | *p* |
|  |  |  |  | LL | UL |  |
| Unadjusted | 242 | 50 | 1.08 | 0.91 | 1.28 | .388 |
| Adjusted | 240 | 50 | 1.09 | 0.91 | 1.30 | .355 |
| Participants included have LTE data recorded at baseline.  The adjusted model included age, sex at birth, ethnicity, site, and medication use. | | | | | | |
